# From valleys to peaks: how climate and environment drive Alpine populations of *Ixodes ricinus*

**DOI:** 10.1186/s13071-026-07590-z

**Published:** 2026-08-04

**Authors:** Alessandro Bellato, Aitor Garcia-Vozmediano, Laura Tomassone, Annibale Biggeri, Dolores Catelan

**Affiliations:** 1 Department of Veterinary Sciences, University of Turin, Grugliasco, Italy; 2 Istituto Zooprofilattico Sperimentale del Piemonte, Liguria e Valle d’Aosta, Turin, Italy; 3 Biostatistics, Epidemiology, and Public Health Unit (UBEP), University of Padua, Padua, Italy

**Keywords:** *Ixodes ricinus*, Spatiotemporal modelling, Bayesian modelling, Alps, Remote sensing

## Abstract

**Background:**

*Ixodes ricinus* is the most widespread tick in Europe and an important vector of pathogens affecting humans and animals. Human cases of tick-borne diseases are rising, reflecting the expansion of *I. ricinus* to higher latitudes and altitudes and the increasing recreational use of Alpine environments. Land-use change, growing wildlife populations, and farmland abandonment are creating new suitable habitats, while climate warming is reducing winter severity at elevation. In the northwestern Italian Alps, *I. ricinus* is now regularly detected above 1600 m. This study investigated environmental and climatic predictors of nymph presence and density in a recently colonized Alpine valley.

**Methods:**

From 2016 to 2019, nymph presence and density were measured six times per year across 44 sites covering an elevation gradient and diverse habitats. Habitat features, wildlife signs, remote-sensing indices, and meteorological variables were recorded or derived. Generalized linear mixed models were used to assess associations with nymph density. A machine-learning Bayesian algorithm was then applied to identify the most influential predictors, accounting for high-dimensionality and collinearity of the predictors set.

**Results:**

Altitude, habitat type, and wildlife presence were associated with nymph presence and density, but their predictive value disappeared in the Bayesian model once climatic and weather variables were included. Global solar irradiation, ground-level relative humidity, soil dryness, and lagged indicators of unfavourable conditions (windy and cold days) consistently predicted nymph density. Terrain descriptors and NDVI-based variables performed poorly, likely due to snow cover and the short warm season typical of Alpine systems.

**Conclusions:**

Short-term weather and fine-scale climatic variables explained variation in nymph density more effectively than broader environmental descriptors within the Bayesian framework, suggesting that colonisation in this Alpine area remains contingent on favourable environmental conditions rather than reflecting fully stable establishment.

**Graphical abstract:**

**Figure:**
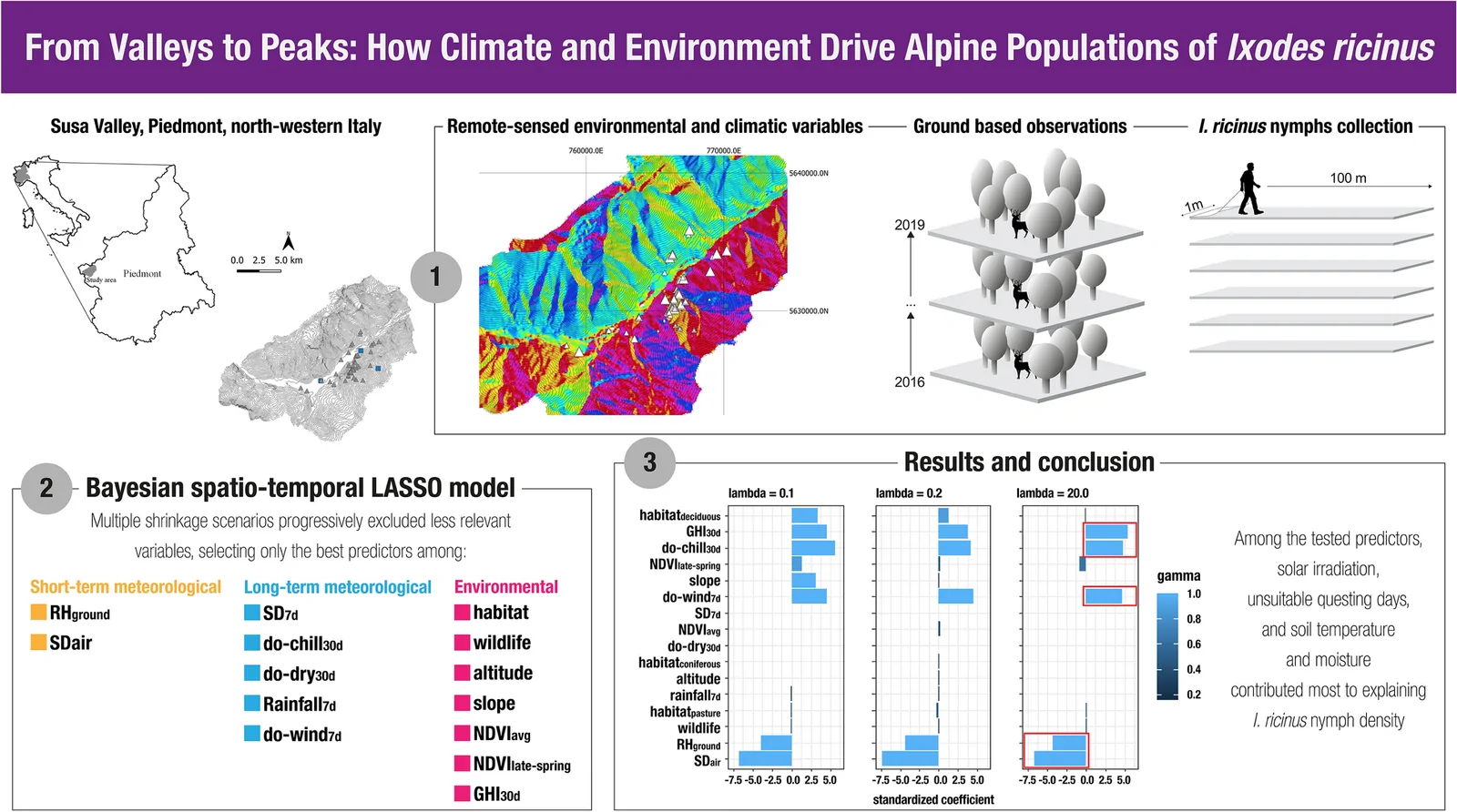

**Supplementary Information:**

The online version contains supplementary material available at https://doi.org/10.1186/s13071-026-07590-z.

## Background

*Ixodes ricinus* is the main vector of tick-borne pathogens affecting humans and animals in Europe [1]. This species is capable of transmitting a wide range of infectious agents, including Lyme borreliosis (LB) spirochaetes, rickettsiales, tick-borne encephalitis virus, and several other pathogens of public and veterinary health relevance. Across Europe, the epidemiology of tick-borne diseases has undergone substantial changes over recent decades, with increasing incidence and expanding geographic distribution associated with environmental and climatic changes [1–3]. In the Piedmont region (north-western Italy), LB case notifications have progressively increased, rising from eight reported cases during 1990–2008 up to 160 cases between 2012 and 2024 [4]. This trend is likely driven by a combination of factors, including the increasing abundance and spatial expansion of *I. ricinus* populations, alongside heightened clinical awareness and improvements in surveillance systems. Several studies conducted across Central and Northern Europe have documented an altitudinal and latitudinal expansion of *I. ricinus* populations over recent decades, largely associated with climate warming, land-use modifications, reforestation processes, and changes in wildlife host communities [1, 5-7]. In Alpine environments, early ecological studies suggested that vegetation cover and climatic constraints limited the distribution of *I. ricinus* to elevations below approximately 1500 m a.s.l. [8]. However, the species has recently been detected above 1700 m [9], and colonization processes have been documented in Alpine areas where the tick was previously considered rare or absent [9].Although historical absence data are frequently limited due to the lack of standardized long-term sampling, these observations are consistent with broader expansion patterns reported across Europe [1, 7, 10]. In particular, Garcia-Vozmediano et al. (2020) [9] reported the establishment of *I. ricinus* populations in areas of the upper Susa Valley (north-western Italian Alps) where previous investigations on wild ungulates had failed to detect the species [11], suggesting an ongoing ecological shift in this Alpine system.

The distribution, abundance and seasonal activity of *I. ricinus* are strongly regulated by climatic and environmental conditions [12–16]. Temperature and humidity are considered particularly important drivers because this species is highly susceptible to desiccation and demands adequate soil and vegetation moisture to survive [8, 17]. Relative humidity at ground level directly influences water balance and physiologic stress in ticks [8, 17], while air saturation deficit strongly affects the questing behaviour by reflecting the drying power of the atmosphere [18]. Perret et al. [19] identified approximately 7 °C as the lower threshold for questing activity, a finding later confirmed by Tagliapietra et al. [20]. Conversely, Hauser et al. [21] observed a reduced host-seeking activity at temperatures exceeding 27 °C or saturation deficit above 6–7 mmHg [20]. Although several studies described non-linear or interacting effects between climatic variables [20, 22, 23], saturation deficit remains a valuable synthetic indicator since it integrates both temperature and relative humidity [18, 21].

Beyond direct climatic constraints, habitat structure and landscape composition may influence *I. ricinus* populations by shaping local microclimatic suitability and host availability [3, 24, 25]. Increasing evidence suggests that broad ecological descriptors such as altitude, slope, vegetation cover, or land-use categories often act as indirect proxies for fine-scale microclimatic conditions regulating tick survival and activity [26–28]. In particular, studies conducted along European mountain gradients have shown that apparent altitudinal patterns of *I. ricinus* may largely reflect underlying thermos-hygrometric conditions. For example, Burri et al. [29] and Morán Cadenas et al. [30] demonstrated that saturation deficit and local temperatures regimes explained non-linear density patterns along Alpine altitudinal gradients, including situations where lower elevations became less suitable because of excessive dryness. Terrain characteristics including aspect, slope, and ruggedness affect solar radiation, moisture retention, and vegetation structure, thereby influencing habitat suitability for both ticks and vertebrate hosts [13, 14]. In fragmented habitats, additional environmental and anthropogenic factors may further modulate tick abundance and persistence [1, 31]. Among vegetation-related indicators, the Normalized Digital Vegetation Index (NDVI) is one of the most investigated predictors of *I. ricinus* density [14, 15, 32–36], partly because vegetation-rich habitats favour the maintenance of humid microclimatic conditions [8, 17, 37]. However, the predictive value of vegetation proxies may itself vary according to preceding meteorological conditions [34], highlighting the importance of jointly evaluating habitat and climatic variables.

Host community composition also plays a central role in shaping *I. ricinus* population dynamics and pathogen circulation. *I. ricinus* feeds on a broad spectrum of vertebrate hosts, including rodents, insectivores, birds, reptiles, and large mammals [38, 39]. Consequently, wildlife abundance and diversity have frequently been incorporated into predictive ecological models [13, 20, 40]. Small mammals and passerine birds represent the main feeding hosts for immature stages and act as important reservoirs for several tick-borne pathogens [22, 38, 41–46], whereas large ungulates are essential for adult tick feeding, reproduction, and dispersal [13, 20, 40].

Host-related factors, however, interact closely with climatic and microhabitat conditions. Tagliapietra et al. [20], for example, showed that saturation deficit primarily regulated questing activity, whereas host abundance contributed more strongly to baseline tick density, suggesting that climatic and host-related drivers may operate at different ecological levels. More broadly, several studies have emphasized that wildlife abundance and habitat composition may be mediated by local climatic suitability and microhabitat conditions rather than acting as fully independent ecological drivers [20, 26, 28].

Nevertheless, important uncertainties remain regarding the relative contribution and hierarchical interplay of broad environmental descriptors and fine-scale climatic variables, particularly in recently colonised Alpine ecosystems characterized by strong environmental heterogeneity. Several studies conducted across European mountain systems have suggested that broad-scale ecological patterns may largely emerge from underlying climatic and microclimatic conditions rather than from altitude or habitat characteristics acting independently [29, 30, 47]. More recently, Wongnak et al. [48] showed that meteorological variables strongly regulate *I. ricinus* phenology and questing dynamics, whereas habitat and landscape descriptors mainly define broader baseline suitability. However, relatively few studies have formally evaluated these relationships within recently colonised Alpine systems, where ecological dynamics may differ from those observed in long-established temperate endemic areas. Previous Alpine investigations have mainly focused on descriptive altitudinal gradients, historical expansion patterns, or broad environmental associations, without explicitly disentangling whether variables such as altitude, habitat type, vegetation cover, or wildlife presence represent independent ecological determinants or indirect surrogates of fine-scale climatic suitability.

Garcia-Vozmediano et al. demonstrated that *I. ricinus* nymph density in the upper Susa Valley was associated with altitude, habitat type, and season [9]. However, these associations were identified using conventional statistical approaches that did not explicitly assess whether broad environmental predictors retained explanatory power once detailed climatic and meteorological variables were simultaneously considered. Moreover, previous analyses did not formally account for the hierarchical structure of the data or the potential spatial and temporal dependence associated with repeated ecological observations. Furthermore, although the occurrence of *I. ricinus* at higher elevations is increasingly documented across Europe, the extent to which these populations represent fully stabilised mountain populations or condition dependent systems regulated by short-term climatic variability remains insufficiently explored [49].

To address these gaps, we analysed previously collected multi-year field data together with newly derived meteorological and remote-sensing variables using a complementary framework integrating mixed-effects models and Bayesian LASSO regression. The mixed-effects models were used to reassess previously reported ecologic associations while accounting for hierarchical dependence and repeated observations, whereas the Bayesian LASSO approach was applied to evaluate the relative contribution of partially correlated broad environmental and fine-scale climatic predictors within high-dimensional ecological setting. We hypothesized that terrain features, vegetation structure, wildlife presence, and meteorological variables jointly influence *I. ricinus* nymph density, but that fine-scale climatic conditions would account for part of the broad ecological associations previously attributed to altitude, habitat or wildlife variables. By leveraging a multi-annual design, this study also provides the opportunity to evaluate interannual variability and the temporal stability of ecological patterns in a recent colonized Alpine system. More specifically, the study aimed to clarify whether the ecological patterns previously described for Alpine *I. ricinus* populations primarily reflect broad environmental structure or more proximal climatic constraints regulating questing dynamics at high elevation.

## Methods

### Study design

This retrospective longitudinal observational study integrates field data from the previous multi-annual survey [9] with meteorological and remote-sensing environmental variables to assess the role of climatic and ecological drivers in shaping tick distribution in high-altitude areas.

### Study area

**Fig. 1:**
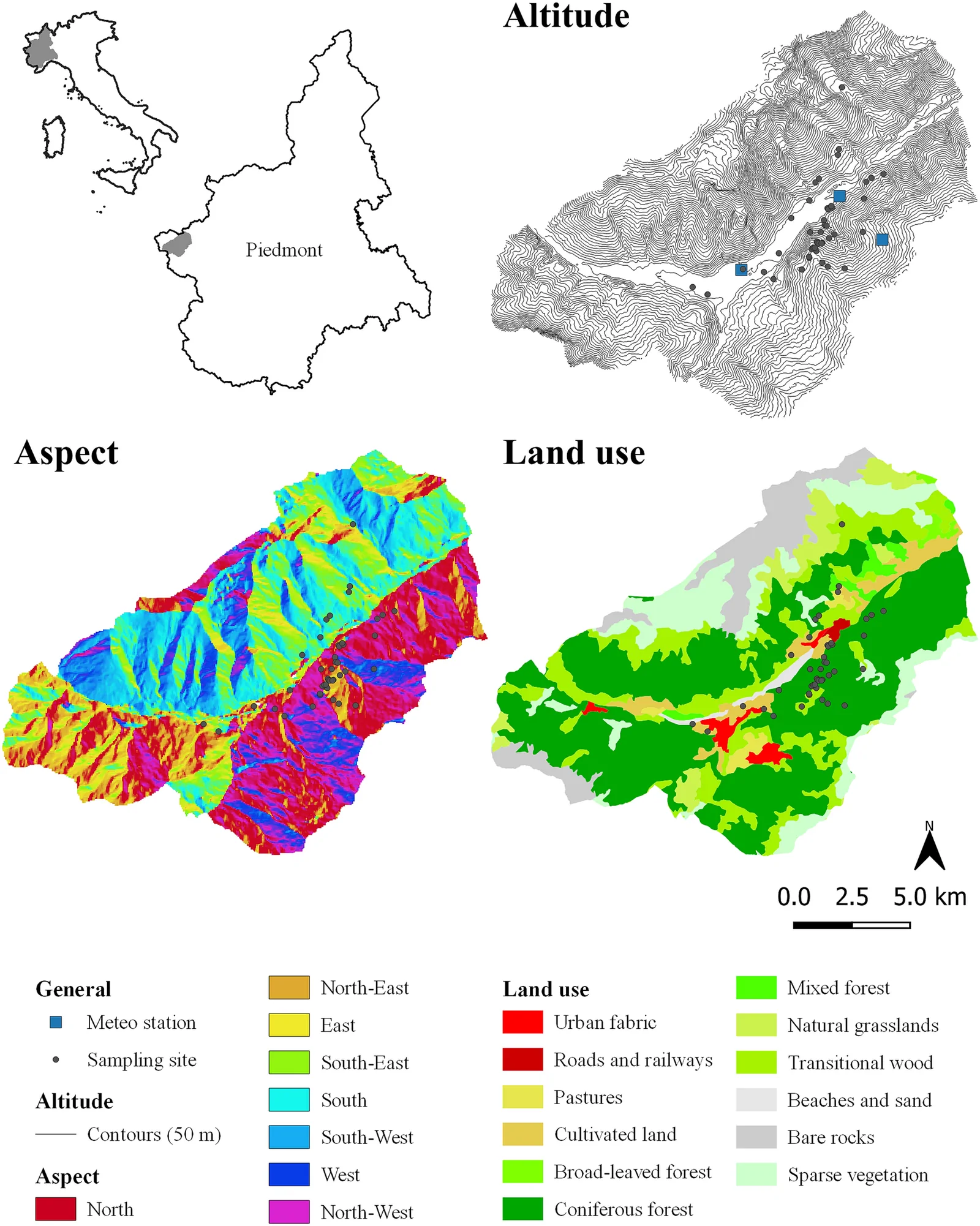
Overview of the study area and the sampling sites. The map illustrates the distribution of the sampling sites across the upper part of the Susa Valley. The location of the meteorological stations is displayed on the “Altitude” thematic map with contours. The “Aspect” thematic map categorizes the aspect of the territory into 8 classes, as calculated from the Digital Terrain Model. The “Land use” thematic map shows land usage in the study area, as obtained from the Corine Land Cover of 2018 (https://land.copernicus.eu/pan-european/corine-land-cover)

The study area is in the upper part of the Susa Valley (Piedmont region), a mountainous area in the western Italian Alps that includes part of the Alpi Cozie Regional Park (45°03′ N, 6°54′ E) (Fig. 1).

Susa Valley stretches westward from the city of Turin to the French border. It is densely inhabited and attracts tourists and hikers throughout the year. From the early 1960s until 2010, maximum temperatures’ records showed an increase of 0.072 °C per year, especially during the cold season and at altitudes above 1600 m a.s.l. [50]. As in other Alpine areas, rainfall in the Susa Valley is concentrated in late spring, summer, and partially in autumn. Snow cover used to be present from late November to April (ARPA Piemonte, Regional Agency for the Protection of the Environment: http://www.arpa.piemonte.it), but it has reduced in recent years [51]. As the altitude increases, deciduous woods gradually mingle with those of conifers until, at higher altitudes, meadows prevail, partly exploited for pastoral use. A large variety of wildlife inhabit the area, including several ungulate species, with Alpine chamois (*Rupicapra rupicapra*) and red deer (*Cervus elaphus*) being the predominant ones (Regional Park rangers, personal communication). For a detailed description of the habitat and wildlife, refer to Garcia-Vozmediano et al. (2020) [9].

A total of 44 sampling sites were purposely selected within the study area based on accessibility and logistical feasibility. Their prevalent aspect was north-east (*n =* 13), followed by east warding slopes (*n =* 9), while the least prevalent aspects were north-west (*n =* 3), west (*n =* 1) and south-west (*n =* 1).

### Tick collection

As described in Garcia-Vozmediano et al. [9], questing *I. ricinus* nymphs’ density of 44 sites (from 959 and 1884 m a.s.l.) was measured monthly, from April 2016 to November 2019, by dragging method. This sampling method is well suited for collecting *I. ricinus* nymphs since they are usually found questing on grass blades that are not as high as shrubs and low branches. No sampling was performed from December to March or even in later months at high altitudes since the snowpack still covered the ground due to adverse weather conditions. Likewise, sampling was avoided during windy or rainy days.

### Local meteorological variables

Temperature and relative humidity were measured both at ground level and 1.5 m above the ground level (T_ground_, RH_ground_, T_air_, and RH_air_, respectively) using a HI9564 thermo-hygrometer (HANNA instruments®, Milan, Italy). From the recorded measurements, air saturation deficit (SD_air_) was calculated using the following equation [52]:

1 $$SD=\left(1-{RH}_{air}\right)\times 4.9463\times {e}^{0.0621\times {T}_{air}}$$

where RH_air_ is measured as a proportion [0,1], and T_air_ is expressed in Celsius degrees (°C).

### Temporal scale

Meteorological variables were summarised over different temporal windows to reflect the heterogeneous time scales at which environmental factors may influence *I. ricinus* ecology. Variables expected to immediately affect questing behaviour were measured at the time of sampling (e.g., relative humidity, saturation deficit), hence hereafter are referred to as concurrent variables. Variables expected to affect questing activity and tick survival over short time spans, such as saturation deficit, precipitation, and wind, were calculated over a 7-day window preceding each transect. Conversely, variables reflecting cumulative environmental stress, including the proportion of days with low temperatures or extreme dryness and the solar irradiance, were summarised over a 30-day window. These time windows were chosen based on biological plausibility and ecological considerations rather than formal optimisation, with the aim of capturing both short-term and medium-term environmental influences on IRN presence and density.

### Meteorological variables

Data were extracted from the public historical meteorology database provided by ARPA Piemonte. The nearest measuring station was selected for each site based on the shortest cartesian distance. Three meteorological stations were selected (Salbertrand: alt. 1010 m; lat. 45.0416 N, long. 6.5338 E; Le Selle: alt. 1980 m; lat. 45.0316 N, long. 6.5502 E; Gad: alt. 1065 m; lat. 45.0234 N, long. 6.5025 E), located at a distance between 77 and 4670 m from the sites. Minimum, maximum, and mean daily temperature and relative humidity (T_min_, T_max_, T_avg_, RH_max_, RH_min_, and RH_avg_, respectively), the daily sum of precipitation, and the mean daily wind speed (V_avg_) were collected from the meteorological station and converted to each transect. Temperatures were adjusted for altitude differences using a fixed lapse-rate approximation, aimed at accounting for large-scale altitude differences and improving comparability between meteorological stations and sampling sites:

2 $${T}_{adj}={T}_{s}-\Gamma \left({A}_{t}-{A}_{s}\right)$$

where T_s_ (T_avg_, T_min_, or T_max_, alternatively) is the temperature in Celsius degrees measured at the meteorological station, A_t_ and A_s_ represent the altitude of the transect and of the station, respectively, and Γ is a location-specific lapse-rate coefficient equal to 0.0056 °C m^−1^. The adjusted mean daily temperature (T_adj_) was used to calculate the mean daily saturation deficit with (1). The SD values for the seven days before sampling were averaged to obtain the mean daily SD for the seven days before sampling (SD_7d_). Daily precipitation values (mm/m^2^) during the same seven-day period were summed to obtain the total rainfall (rainfall_7d_).

The days deemed unsuitable for *I. ricinus* questing were defined as follows: days of chilling (do-chill) when the T_max_ did not exceed 7 °C [19]; days of drying (do-dry) when the minimum saturation deficit $\left( {SD_{{min}} = \left( {1 - RH_{{max}} } \right) \times 4.9463 \times e^{{0.0621 \times T_{{min}} }} } \right)$) was higher than 7.3 mmHg [21]. Days of wind (do-wind) were defined as those when the V_avg_ exceeded 5.5 m/s, the threshold separating gentle from moderate breeze according to the Beaufort scale (https://www.weather.gov/mfl/beaufort). Additionally, do-chill and do-dry proportions were calculated over a 30-day period before each transect (do-chill30d, and do-dry30d, respectively), and the do-wind proportion over a 7-day period (do-wind7d). The time window used to calculate the change in each variable was arbitrarily selected to ensure sufficient variability across sites, in accordance with the ecological and phenological characteristics of the vector.

### Environmental variables

Based on the dominant plant species and vegetation structure, the habitat was categorized into: (1) open areas, including pastures with herbaceous and shrub species; (2) coniferous woods, including fir, larch, spruce, and pine; and (3) deciduous woods, including birch, ash, willow, poplar, and beech.

The presence of faeces, footprints, and other traces was recorded for nine different species/animal groups: red deer (*C. elaphus*), roe deer (*Capreolus capreolus*), chamois (*R. rupicapra*), wild boar (*Sus scrofa*), fox (*Vulpes vulpes*), wolf (*Canis lupus italicus*), mustelids (fam. *Musteliadae*), hare (*Lepus europaeus*), and squirrel (*Sciurus vulgaris*). The proportion of transects where wildlife traces were observed was calculated for each site over the entire study period.

Normalized Digital Vegetation Index (NDVI) raster images (with a spatial resolution of 30 m × 30 m, and a temporal resolution of 16 days) were obtained from NASA MODIS images captured from March 2016 to November 2019 (https://modis.gsfc.nasa.gov/data/dataprod/mod13.php). Geocoded images were imported into QGIS, pixels with low accuracy due to cloud coverage were excluded, and no further geometric correction was applied. NDVI values were sampled using the coordinates of each site by *sample raster values* QGIS tool, and then their monthly averages were calculated (NDVI_avg_). Late-spring NDVI (NDVI_late-spring_) was calculated for each site by averaging the values recorded from 15th April to 21st June during the study period, as described by Rosà et al. [15]. Since NDVI is the ratio between the near infrared (NIR) and visible radiation (VIS), as described in (3), it ranges from −1 to + 1.

3 $$NDVI=\frac{\left(NIR-VIS\right)}{NIR+VIS}$$

The highest values correspond to a dense green leaves’ coverage. When green leaves are absent, the values hover around 0, whereas other non-vegetational surfaces yield negative values.

Altitude, slope, and aspect were obtained from the DTM of the Piedmont region (Istituto Superiore per la Protezione e la Ricerca Ambientale, ISPRA) with a spatial resolution of 50 m × 50 m. The altitude was determined using the *sample raster values* tool and expressed in metres above sea level (m a.s.l.). Slope (i.e., the steepness of an inclined surface) was computed by a dedicated *raster calculator* function in QGIS and expressed as a ratio [0,1] of the rise over the run. Aspect (i.e., the geographic orientation of the slope), was calculated using a dedicated *raster calculator* function and expressed as a numerical value between 0 and 360 degrees, measured clockwise from the north. It was categorized in eight sectors, namely North [0°, 22.5°), [337.5°, 360°]; North-East [22.5°, 67.5°); East [67.5°, 112.5°); South-East [112.5°, 157.5°); South [157.5°, 202.5°); South-West [202.5°, 247.5°); West [247.5°, 292.5°); and North-West [292.5°, 337.5°).

Global horizontal irradiation (GHI) is defined as the sum of direct and diffuse solar irradiation [53]. It is influenced by latitude, slope, and aspect. Daily GHI was obtained as raster images from the SoDa Helioclim-3 Archives (Solar radiation Data, http://www.soda-pro.com/web-services/radiation/helioclim-3/). For each transect, GHI values were sampled using *sample raster values* tool, whereas pixels with cloud coverage exceeding 20% were excluded. Daily measures of the 30 days before each transect were averaged (GHI_30d_).

QGIS version 3.4.4-Madeira (QGIS Project, GNU General Public License, https://qgis.org/en/site) was used to gather and manage data of different nature into a single geographical database.

### Data analysis

The analytical framework adopted in this study consisted of two complementary modelling stages addressing different inferential objectives. First, generalized linear mixed models (GLMMs) were used to reassess previously reported ecological associations while accounting for repeated observations and hierarchical site-level structure. Second, Bayesian Lasso regression was applied to investigate the relative contribution of fine-scale meteorological, environmental and vegetation-related predictors under conditions of multicollinearity and high predictor dimensionality.

#### Descriptive statistics

This study focused on *I. ricinus* nymphs’ (IRN) presence (i.e., the finding of at least one *I. ricinus* nymph in a 100 m^2^ transect) and IRN density (i.e., the number of *I. ricinus* nymphs collected over a 100 m^2^ transect). The association between IRN density and categorical variables was assessed using the Kruskal–Wallis rank-sum test. The association between IRN presence and categorical variables was assessed using a *χ*^2^ test after verification of expected cell counts.

Garcia-Vozmediano and colleagues (2020) [9] investigated the association of altitude, habitat type, and the presence of wildlife with IRN presence and density. In the present study, these associations were first reassessed using Generalized Linear Mixed Models (GLMMs) [54] to account for the hierarchical structure of the data and to provide a frequentist reference framework against which subsequent Bayesian analyses could be interpreted. In addition to the variables considered by Garcia-Vozmediano et al. [9], an extended set of environmental and meteorological predictors included in the Bayesian modelling framework was fitted in supplementary GLMMs to enable diagnostic assessment of spatial and temporal dependence and to ensure comparability between analytical approaches. For IRN presence, a binomial distribution with a logit link function was assumed, whereas IRN density was modelled using a Poisson distribution with a log link function. All models included fixed effects (X_ij_) for altitude, habitat type, presence of wildlife, season and the additional environmental and meteorological covariates also considered in the Bayesian analysis. A site-specific random intercept (u_i_) was incorporated to account for repeated observations within sampling sites and between-site heterogeneity.

The model for IRN presence was specified as:

4 $$\mathrm{logit}\left({P}_{ij}\right)=\alpha +{u}_{i}+\sum_{k=1}^{p}{\beta}_{k}{X}_{ij}^{k}$$

where P_ij_ denotes the probability of IRN presence at transect j within the site i, and β_k_ are the fixed-effects coefficients.

The model for IRN density was specified as:

$$\log\left({\lambda}_{ij}\right)=\alpha +{u}_{i}+\sum_{k=1}^{p}{\beta}_{k}{X}_{ij}^{k}$$ 5﻿

where λ_ij_ denotes the expected IRN density.

The fitted mixed-effects models were used to characterise site-level heterogeneity and to explore residual spatio-temporal structure. Between-site clustering was quantified through the variance of the random intercepts and by calculating the intraclass correlation coefficient (ICC) [55]. For the binomial model (4), the ICC was computed assuming a logistic distribution:

The fitted mixed-effects models were used to characterize site-level heterogeneity and to explore residual spatio-temporal structure. Between-site clustering was quantified through the variance of the random intercepts and by calculating the intraclass correlation coefficient (ICC) [55]. For the binomial model (4), the ICC was computed assuming a logistic distribution:

6 $$ICC=\frac{{\sigma}_{u}^{2}}{{\sigma}_{u}^{2}+\frac{{\pi }^{2}}{3}}$$

where σ^2^_u_ represents the variance of the site-level random intercepts. For the Poisson model (5), the ICC was calculated as:

7 $$ICC=\frac{{\sigma}_{u}^{2}}{{\sigma}_{u}^{2}+{\sigma}_{se}^{2}}$$

where $${\sigma}_{se}^{2}=\log\left(1+\frac{1}{\mu }\right)$$.

Residual spatial structure was subsequently evaluated using Moran's I statistics under alternative k-nearest-neighbour weighting schemes and by visual inspection of empirical variograms. Temporal dependence was assessed using site-specific autocorrelation (ACF) and partial autocorrelation (PACF) functions of model residuals and, for IRN density, by comparing models with and without an explicit AR(1) correlation structure. Multicollinearity among predictors was evaluated using variance inflation factors (VIFs) and pairwise Spearman correlations.Temporal dependence was assessed using site-specific autocorrelation (ACF) and partial autocorrelation (PACF) functions of model residuals and by comparing models with and without an explicit AR(1) correlation structure. Multicollinearity among predictors was evaluated using the variance inflation factors (VIFs) and pairwise Spearman correlations.

Finally, it should be emphasised that these extended GLMMs were not intended as a variable-selection procedure. Rather, they provided a complementary frequentist description of associations and residual dependence, whereas identification of the most stable and informative predictors was subsequently achieved within the Bayesian LASSO framework through coefficient shrinkage and penalisation.

#### Selection of additional variables

In addition to the variables already analysed in Garcia-Vozmediano et al. (2020) [9] and included in the GLMM framework above, a set of additional environmental and meteorological predictors was included and statistically described. Pairwise Spearman correlations and generalized variance inflation factors (GVIF) were calculated to assess multicollinearity among candidate predictors prior to model fitting (Figure S1, Table S7, 8). On the basis of both statistical dependence and biological plausibility, candidate variables were identified for inclusion in the final model, thereby reducing the risk of overfitting while preserving ecological interpretability.

The selected variables were grouped into concurrent variables (RH_ground_ and SD_air_); short-term meteorological variables (do-wind_7d_, SD_7d_, and rainfall_7d_); medium-term meteorological variables (do-chill_30d_, do-dry_30d_); and environmental variables (habitat, wildlife traces, altitude, slope, NDVI_avg_; NDVI_late-spring_, and GHI_30d_).

Table 1 summarises the variability in space and time for each variable included in the model.

**Table 1:** List of selected variables included in the model to describe the distribution of *I. ricinus* nymphs

Covariate	Category	Varies in space	Varies in time	Time window	Type	Unit
RH_ground_	Concurrent meteorological	By site	By day	No	Continuous	%
SD_air_	Concurrent meteorological	By site	By day	No	Continuous	mmHg
SD_7d_	Short-term meteorological	By site	By day	7 days	Continuous	mmHg
Rainfall_7d_	Short-term meteorological	By station	By day	7 days	Continuous	mm/m^2^
Do-wind_7d_	Short-term meteorological	By station	By day	7 days	Continuous	%
Do-chill_30d_	Medium-term meteorological	By site	By day	30 days	Continuous	%
Do-dry_30d_	Medium-term meteorological	By site	By day	30 days	Continuous	%
Habitat	Environmental	By site	No	–	Categorical	1–4
Wildlife traces	Environmental	By site	No	–	Continuous	%
Altitude	Environmental	By site	No	–	Continuous	m a.s.l
Slope	Environmental	By site	No	–	Continuous	Ratio [0, 1]
NDVI_avg_	Environmental	By site	By month	~ 30 days	Continuous	Ratio [− 1, 1]
NDVI_late-spring_	Environmental	By site	No	~ 30 days	Continuous	Ratio [− 1, 1]
GHI_30d_	Environmental	By site	By day	30 days	Continuous	Wh/m^2^

#### Bayesian spatio-temporal least absolute shrinkage and selection operator (LASSO) model

To investigate the joint effects of multiple, potentially correlated environmental and meteorological variables on IRN density, we adopted a Bayesian spatio-temporal LASSO framework. The Bayesian variable-selection framework was restricted to IRN density rather than IRN presence because abundance data provide greater ecological sensitivity for investigating fine-scale environmental modulation of questing activity. While occurrence data mainly reflect broad habitat suitability and establishment, density measurements more directly capture temporal fluctuations associated with short-term meteorological and microclimatic conditions. This approach was chosen because it allows simultaneous variable selection and parameter estimation in high-dimensional settings, while mitigating multicollinearity and reducing the risk of overfitting. The extended environmental predictor set included multiple correlated climatic, vegetation, and topographic variables, making coefficient interpretation and variable selection challenging within a conventional mixed-effects framework. The Bayesian formulation provides full posterior distributions for model parameters, enabling uncertainty quantification and probabilistic interpretation of variable inclusion. In addition, it allows all candidate predictors to be retained within a single probabilistic framework, enabling posterior inclusion probabilities to be evaluated, too, rather than relying on fixed selection thresholds. This provides a more flexible and transparent basis for assessing variable relevance under uncertainty.

Let Y_ij_ be the IRN density in the i-th site (i = 1, …, 29) and j-th sampling (j = 1, …, 24). The likelihood is assumed to be Poisson with parameter (θ_ij_). We assumed a log-linear model for the mean. In particular:

8 $$\log\left({\theta}_{ij}\right)=\alpha +{u}_{i}+{\nu}_{i}+{t}_{j}+{\beta}_{1}{\gamma}_{i}{X}_{ij}^{i}+...+{\beta}_{k}{\gamma}_{k}{X}_{ij}^{k}+...+{\beta}_{p}{\gamma}_{p}{X}_{ij}^{p}$$

where **X**_**ij**_ is the vector of p variables (k = 1, …, p) for the i-th site and j-th time and γ is a vector of binary indicators that indicates whether variables are included in the model (with γ_k_ = 1) or not (with γ_k_ = 0).

The a priori distributions are: β_k_ ~ Double Exponential (0, 1/δτ), τ ~ Gamma (0.001,0.001), γ_k_ ~ Bernoulli (0.5), where τ identifies the precision (i.e., the inverse of the variance) and δ a tuning parameter.

Different values of the shrinkage parameter δ control the degree of penalizations applied to regression coefficients. Smaller values of δ correspond to weaker shrinkage and large variance, thus introducing low information in the Bayesian model (i.e., more permissive model). Vice versa, larger values of δ impose stronger penalisation, shrinking non-informative coefficients towards zero by introducing strong a priori on β’s close to zero. To evaluate the robustness of the results, the model was fitted under five values of δ (0.05, 0.1, 0.2, 5.0, and 20.0), representing increasing levels of shrinkage. This approach was used as a sensitivity analysis to assess the stability of selected predictors across qualitatively different penalisation regimes, rather than to exhaustively explore the parameter space in search of an optimal value. The term α represents the intercept a priori modelled as improper uniform. The term *u*_*i*_ is a spatial random effect that considers that areas close to each other tend to behave similarly. Their joint distribution may be expressed as u ∼ MVN(µ, σ^2^_u_ Σ) where MVN stays for Multivariate Normal distribution, µ is the mean vector (usually equal to 0), σ^2^_u_ > 0 controls the overall variability (*λ*_*u*_ = 1/ σ^2^_u_) of the u_i_ and Σ is an N × N positive definite matrix. A parametric form for the elements of the matrix was assumed. In particular Σ_ik_ = exp[-(φ d_ik_)^ξ^] where d_ik_ is the distance between sites i and k a parametric form for the matrix elements may be assumed. In this case, a common assumption is k = 1, φ > 0 controls the rate of decline of correlation with distance and ξ ∈ (0, 2] controls the amount of spatial smoothing [56]. The term φ is chosen to get almost zero correlation at the maximum distance (11 km) and 0.33 at the minimum distance (1.5 km). The term t_j_ is the temporal random effect modelled a priori as Gaussian conditional autoregressive of order 2: where is the mean of the (t-2)-th and (t + 2)-th terms, is the precision and *n*_t_ the number of adjacencies. The term *v*_*i*_ is the nugget effect modelled a priori as Normal (*0, λ*_*v*_). The hyperprior distributions of the precision parameters *λ*_*v*_, *λ*_*u*_ are assumed to be Gamma (*0.5, 0.0005*).

For robustness, a subset of transects was selected to ensure a sufficient number of repeated observations per site. Specifically, the model was restricted to 29 sites with more than 15 transects each, for a total of 724 transects. This selection reflects the focus of the analysis on IRN density under conditions where tick populations are established. Sites with fewer observations were typically characterised by consistently absent or sporadic tick detections across the study period. While informative for presence/absence analyses, such sites provide limited information for modelling density variation and may reduce model stability in count-based frameworks. The applied threshold therefore ensured both adequate temporal replication and the inclusion of sites where density dynamics could be meaningfully assessed. All continuous variables were standardized.

Statistical analyses were performed using R software (version 4.0.3, https://cran.r-project.org) and OpenBUGS (version 3.2.3, https://www.mrc-bsu.cam.ac.uk/software/bugs/openbugs).

## Results

**Fig.2:**
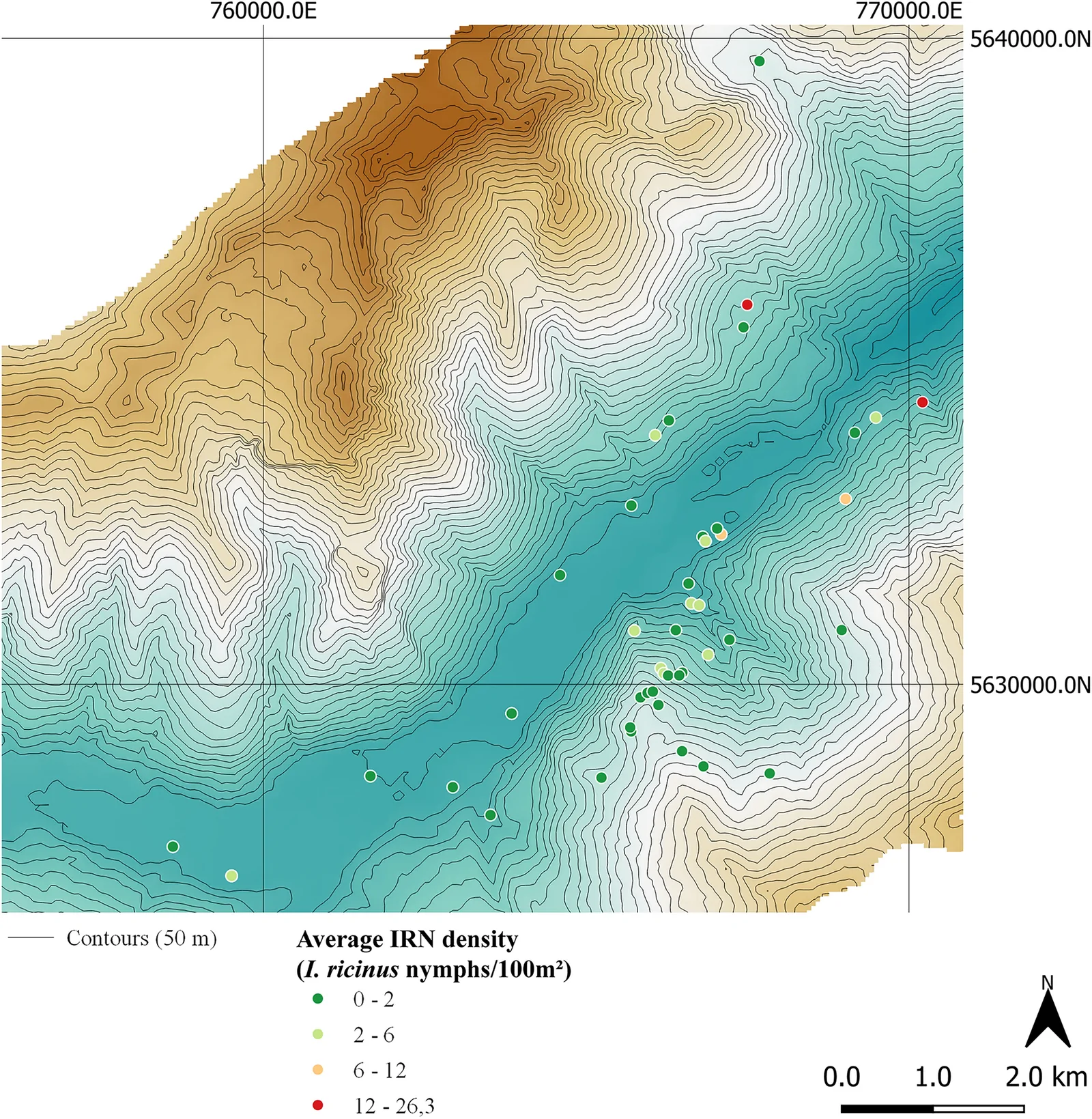
Average *I. ricinus* nymph density per site. The map of the study area represents the average *I. ricinus* nymphs’ density over 100 m^2^ transects compared to altitude. The color of the dots (from green to red) corresponds to the average nymph density as shown in the legend [Reference system: WGS 84 / UTM zone 32N]. Particular of Susa Valley, Italian north-western Alps. 2016–2019

Out of 872 transects sampled, 2388 IRN were collected between April 2016 and November 2019. Overall, a prevalence of 47.2% (95%CI: 43.9–50.6%) transects were infested, and an IRN density of 2.7 IRN/100 m^2^ was recorded (95%CI: 2.6–2.9 IRN/100 m^2^). Differences in IRN density across slope aspects were observed, with higher values on south-east and north-west facing slopes. However, these patterns should be interpreted with caution, as certain slope exposures were underrepresented in the sampling design (Fig. 2).

**Fig.3:**
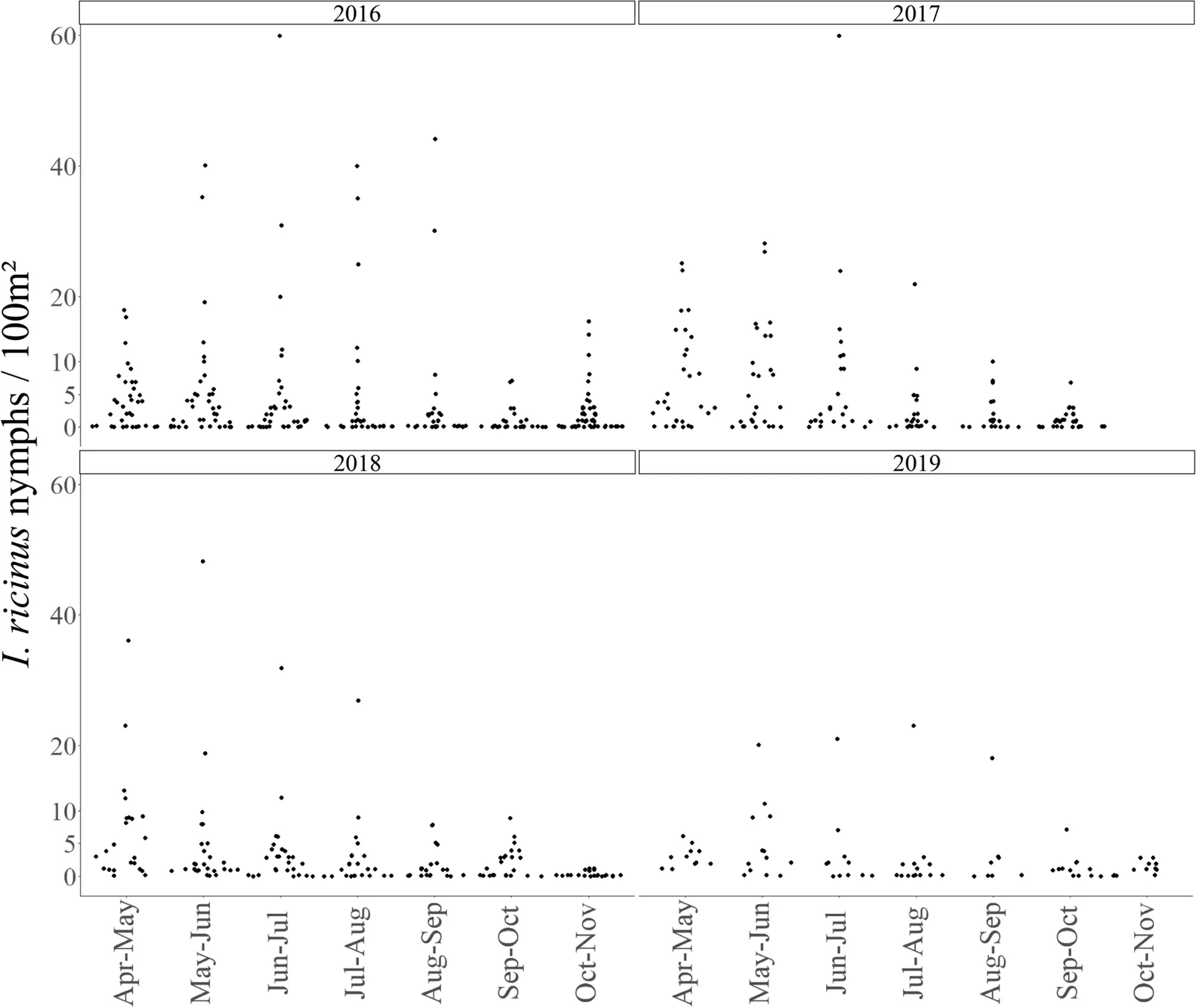
Sina plots display *I. ricinus* nymphs’ density by year and month. Susa Valley, Italian north-western Alps. 2016–2019

Descriptive analysis of IRN presence and density in relation to season, altitude, habitat type and animal traces were consisted with those previously reported [9], and are summarized here for completeness. IRN presence varied across months, altitude, habitat type, and animal traces, showing higher values in spring, at intermediate altitudes (1200–1400 m a.s.l.), and in deciduous woodland habitats, particularly in the presence of wild animals. Similar patterns were observed for IRN density, which peaked between April and June and was highest at intermediate altitudes and in deciduous woods. No significant variation across years was detected for either presence or density. Detailed results of the descriptive analyses are reported in Tables S1–S5. Observed IRN density across months and years is shown in Fig. 3, highlighting seasonal patterns and interannual variability.

The results of the model for IRN presence and IRN density including all variables described in Garcia-Vozmediano et al. [9] are presented in Table 2.

**Table 2:** Estimated effects of altitude, habitat, presence of animals, and seasonality on *Ixodes ricinus* nymphs (IRN) presence and density

Variable category	Variable	IRN presence		IRN density	
		OR	95%CI	IRR	95%CI
	Intercept	3.26	2.00–5.41	4.15	3.71–4.64
Altitude	≤ 1400 m	1 (ref.)	–	1 (ref.)	–
	> 1400 m	0.34	0.24–0.48	0.5	0.45–0.55
Habitat	Coniferous woods	1 (ref.)	–	1 (ref.)	–
	Deciduous woods	2.37	1.45–3.97	2.49	2.27–2.72
	Open areas	0.27	0.19–0.39	0.33	0.29–0.37
Animal presence	No traces	1 (ref.)	–	1 (ref.)	–
	Livestock	1.08	0.58–1.97	1.15	0.96–1.38
	Wildlife	1.88	1.31–2.69	1.71	1.57–1.87
Season	Apr–May	1 (ref.)	–	1 (ref.)	–
	May–Jun	1.04	0.58–1.85	1.16	1.03–1.30
	Jun–Jul	0.76	0.43–1.35	1.01	0.89–1.15
	Jul–Aug	0.35	0.19–0.62	0.65	0.56–0.74
	Aug–Sep	0.26	0.14–0.47	0.45	0.38–0.53
	Sep–Oct	0.28	0.16–0.50	0.25	0.21–0.31
	Oct–Nov	0.23	0.13–0.41	0.24	0.19–0.29

The GLMM revealed substantial between-site heterogeneity for both IRN presence and density, as indicated by the random-intercept variance (σ^2^_u_ = 3.5 and 2.23, respectively) and the intraclass correlation coefficient (ICC = 0.48 for IRN presence and 0.81 for density), supporting the presence of strong site-level clustering and non-independence among repeated observations. However, residual spatial diagnostics yielded contrasting results between the two responses. Moran's I statistics computed using k-nearest neighbour weighting schemes did not indicate significant residual spatial structure for IRN presence (Table S9), whereas significant positive Moran's I values across several neighbourhood definitions were observed for IRN density (Table S11), suggesting the presence of residual spatial dependence in abundance patterns. Exploratory temporal diagnostics similarly differed between responses. For IRN presence, lag-1 correlations were weak and inconsistent among sites, and an explicit AR(1) correlation structure did not improve model fit (Figure S2–S4A–C; Table S10). In contrast, several sites exhibited short-term temporal dependence in IRN density (Figure S5-S7A-C), and likelihood-ratio tests supported the inclusion of an AR(1) structure, indicating that temporal persistence in tick abundance may occur at local scales (Table S12). Together, these findings supported the inclusion of random effects to account for site-level clustering and motivated the subsequent Bayesian spatio-temporal modelling framework for IRN density.

**Fig. 4:**
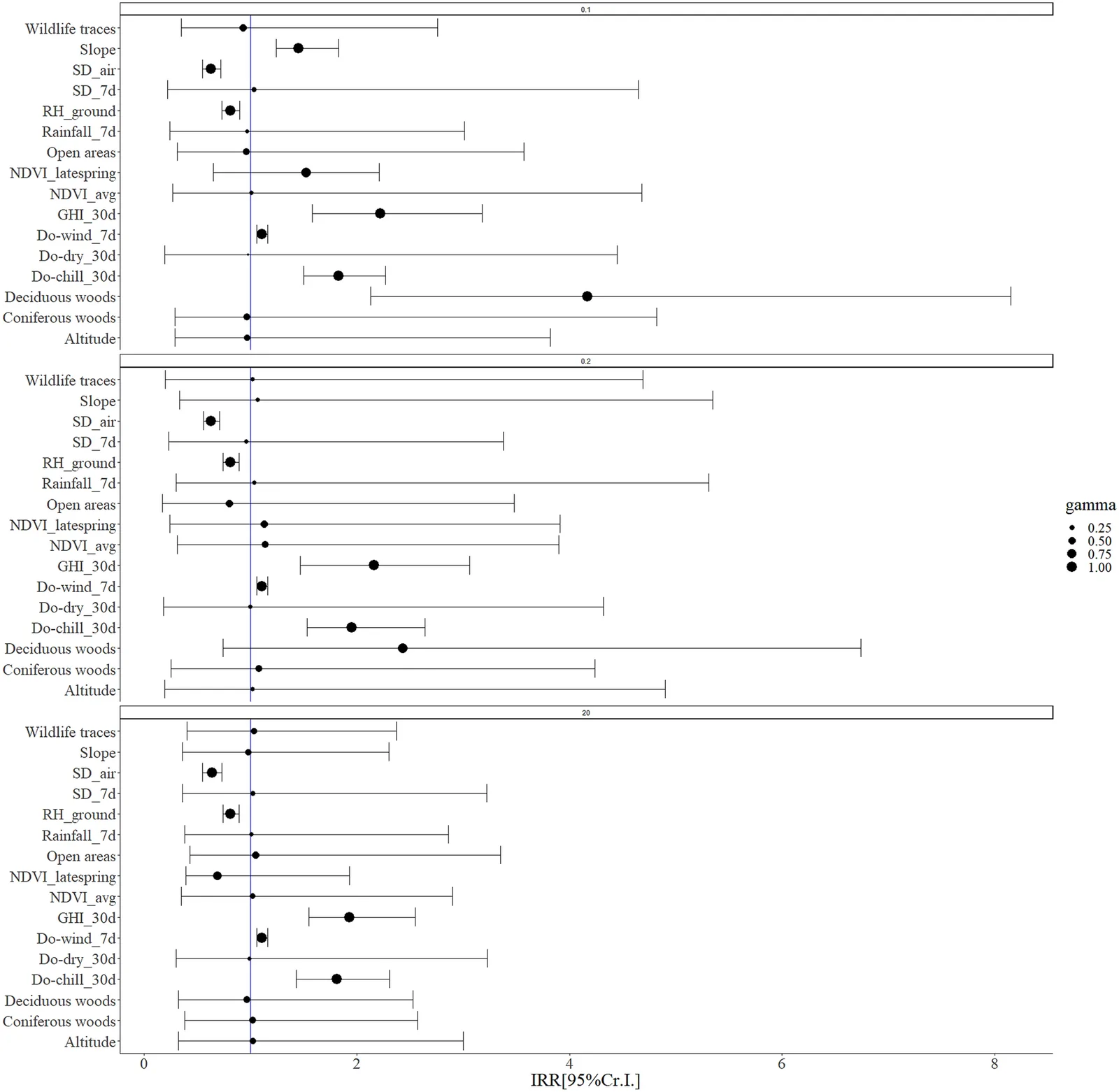
Incidence rate ratios of the sixteen variables by shrinkage level. The point estimates are reported along with their 95% Credibility Intervals [95%Cr.I.]. The blue line represents the null value (IRR = 1.0). The size of the dots is proportional to the gamma value

The Bayesian LASSO analysis revealed that IRN density is shaped by a combination of environmental and meteorological drivers acting at different temporal scales (Fig. 4).

The Bayesian LASSO was used to model IRN density in a subset of 29 sampling sites using 16 variables (habitat was categorized into three dummy variables). In addition to the environmental and biological predictors previously described (altitude, habitat type, season, and animal traces), this model was extended to include a set of microclimatic, topographic, and vegetation-related covariates (Tables S6; Figure S1). Rather than interpreting each covariate individually, the focus was on the overall contribution of this extended covariate set to the model’s ability to explain IRN density, while accounting for spatial and temporal dependence. The Bayesian LASSO analysis revealed that IRN density is influenced by a combination of environmental and meteorological drivers acting at different temporal scales (Fig. 4).

Across increasing levels of shrinkage, a consistent core of predictors remained associated with IRN density, indicating robust effects. In particular, medium-term meteorological variables showed the strongest and most stable associations consistently positively associated with IRN density, and effect size remaining significant even under the highest shrinkage level.

In contrast, concurrent microclimatic conditions showed a negative association with IRN density, as higher ground-level relative humidity (RH_ground_) and air saturation deficit (SD_air_) were consistently associated with reduced IRN counts.

Several environmental and vegetation-related variables, including slope, late-spring NDVI, and deciduous woodland habitat, were only associated with IRN density at low shrinkage levels, suggesting weaker or context-dependent effects that did not persist under stronger regularization. Other predictors, including altitude and short-term meteorological variables, showed little or no association even at minimal shrinkage. Overall, the model highlights a clear distinction between a limited set of robust predictors and a larger group of variables with unstable or negligible effects (Table 3, S13).

**Table 3:** Incidence rate ratio [IRR], 95% credibility interval [95% Cr.I.] and gamma value for each covariate at the three levels of shrinkage

	IRR	δ = 0.1	γ	IRR	δ = 0.2	γ	IRR	δ = 20.0	γ
		95% Cr.I			95% Cr.I			95% Cr.I	
Wildlife traces	0.93	0.35–2.76	0.48	1.02	0.20–4.69	0.21	1.03	0.40–2.37	0.36
Slope	1.45	1.24–1.83	1	1.07	0.33–5.35	0.20	0.98	0.36–2.30	0.35
SD_air_	0.63	0.55–0.72	1	0.63	0.56–0.71	1	0.64	0.55–0.73	1
SD_7d_	1.03	0.22–4.65	0.22	0.96	0.23–3.38	0.20	1.02	0.36–3.22	0.24
RH_ground_	0.81	0.73–0.90	1	0.81	0.74–0.89	1	0.81	0.74–0.89	1
Rainfall_7d_	0.97	0.24–3.01	0.17	1.04	0.30–5.31	0.18	1.01	0.38–2.86	0.20
Habitat: open areas	0.96	0.31–3.57	0.43	0.8	0.17–3.48	0.52	1.05	0.43–3.35	0.46
NDVI_late-sping_	1.52	0.65–2.21	0.88	1.13	0.24–3.91	0.45	0.69	0.39–1.93	0.64
NDVI_avg_	1.01	0.27–4.68	0.21	1.14	0.31–3.90	0.40	1.02	0.35–2.90	0.26
GHI_30d_	2.22	1.58–3.18	1	2.16	1.47–3.06	1	1.93	1.55–2.55	1
Do-wind_7d_	1.11	1.06–1.16	1	1.11	1.06–1.16	1	1.11	1.06–1.16	1
Do-dry_30d_	0.98	0.19–4.45	0.12	1.00	0.18–4.32	0.18	0.99	0.30–3.23	0.16
Do-chill_30d_	1.83	1.50–2.27	1	1.95	1.53–2.64	1	1.81	1.43–2.31	1
Habitat: deciduous wood	4.17	2.13–8.15	1	2.43	0.74–6.74	0.89	0.97	0.32–2.53	0.39
Habitat: coniferous wood	0.97	0.29–4.82	0.39	1.08	0.25–4.24	0.38	1.02	0.38–2.57	0.42
Altitude	0.97	0.29–3.82	0.36	1.02	0.19–4.90	0.18	1.02	0.32–3.00	0.32

Moderate discrepancies between inclusion probabilities (γ) and corresponding credibility intervals were observed for a limited number of variables. In these cases, the direction of the association was supported by the model, but the uncertainty around effect size remained high, reflecting limited information in the data.

**Fig. 5:**
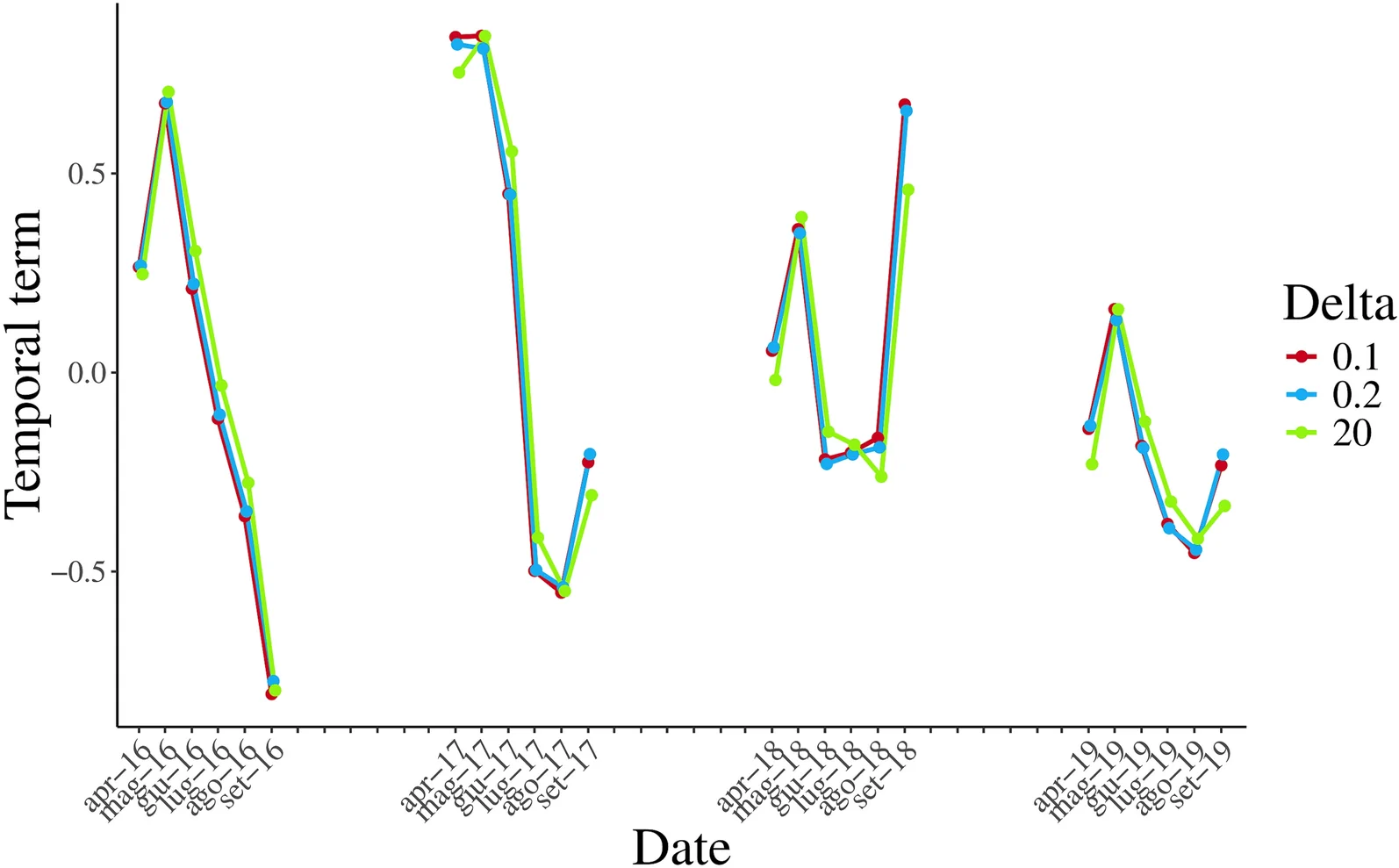
Temporal random term of the LASSO Bayesian model by different levels of shrinkage

The temporal trend captured the main seasonal pattern of IRN density with a consistent spring peak across years and all shrinkage levels, indicating that seasonality represents a stable and reproducible component of tick dynamics over the multi-year study period. On the other hand, autumn dynamics showed greater interannual variability: a late-season increase observed in November 2016 was not fully reproduced by the model, suggesting less stable secondary peaks (Fig. 5).

## Discussion

Consistent with previous findings, IRN presence and density were associated with altitude, habitat type, and wildlife presence, confirming that deciduous woodlands below 1500 m provide favourable conditions for the survival of both adult and immature stages of *I. ricinus* in Alpine environments [8, 9, 18, 13]. The infestation of north-western Alpine areas has been facilitated by the expansion of wooded land and the increase in wild ungulate populations following land-use changes and agricultural abandonment [57–59], while the detection of all developmental stages at higher altitudes suggests an upward expansion likely favoured by climate change [1, 9, 60]. These findings are consistent with broader European evidence documenting the progressive colonization of higher elevations by *I. ricinus* in response to changing climatic and ecological conditions [6, 60-66].

The associations identified in this study, obtained using a different modelling framework, support the robustness of previously identified ecological patterns. In addition, the models revealed spatial and temporal dependence in the observations. In particular, the intraclass correlation coefficient for IRN density was high (ICC = 0.81), indicating that a substantial proportion of variability was attributable to differences between sampling sites and pointing to strong site-specific effects not fully captured by the measured predictors. This residual spatial structure may reflect unmeasured ecological heterogeneity operating at fine spatial scale, including differences in vegetation stratification, litter depth, soil moisture retention, micro-topography, local host community composition, and microhabitat buffering capacity, all of which are known to influence tick survival and questing behaviour[25, 67, 70 –]. Fine-scale environmental heterogeneity has also been identified as an important determinant of local tick abundance in other European systems [68, 70]. Similar findings have been reported in fragmented temperate forest systems, where local habitat buffering and microhabitat structure may outweigh broader climatic conditions in explaining local tick abundance [67]. In Alpine environments, these effects may be further amplified by steep environmental gradients occurring over short distances, generating highly heterogeneous local suitability conditions even within the same broad habitat category. The magnitude of the ICC therefore suggests that the predictors included in the Bayesian model should not be interpreted as exhaustive determinants of IRN density, but rather as variables explaining temporal fluctuations in tick activity conditional on substantial underlying site-level structure. In this context, the use of spatio-temporal Bayesian framework was particularly important because it explicitly accounted for structured dependence and reduced the risk of attributing persistent spatially heterogeneity to fixed environmental effects. Accordingly, the estimated fixed effects should be interpreted as reflecting the influence of climatic and meteorological drivers after accounting for strong site-specific ecological differences that remain only partially measurable at the scale of the available environmental covariates.

Climate and weather shape habitat distribution according to elevation and topography, while habitat in turn constrains wildlife movements and residence times. These variables therefore form interdependent ecological layers and should be regarded as competing explanations rather than as alternative predictors of the same ecological gradient operating at different spatial and temporal scales. In this context, broad environmental descriptors such as altitude, habitat type, or wildlife presence may capture aggregated or coarse-scale effects, while also acting as proxies that partially mask the contribution of finer-scale meteorological and climatic drivers. Accordingly, while these variables were associated with IRN presence and density in GLMMs, their effects were not retained in the Bayesian model once fine-scale meteorological variables were included. This suggests that part of the variation explained by broad environmental descriptors may also be captured by more proximal climatic predictors. Under this framework, the Bayesian model preferentially retained the finer-scale climatic variables, indicating that they explained the observed variation more efficiently, whereas altitude, habitat and wildlife may represent broader ecological descriptors of the same underlying processes. This does not imply that habitat or wildlife are ecologically irrelevant but rather suggests that part of their apparent effect on IRN density may operate indirectly through local climatic suitability and microhabitat buffering. This interpretation is consistent with studies conducted along Alpine gradients, where saturation deficit and local temperature regimes explained non-linear altitudinal patterns of *I. ricinus* density and activity [29, 30]. Similarly, Wongnak et al. (2022) [48] showed that meteorological variables strongly regulate questing phenology, whereas habitat and landscape descriptors mainly define baseline environmental suitability.

Among the climatic predictors retained in the final model, GHI_30d_ emerged as a robust predictor of IRN density, in line with recent findings [59, 71]. This metric integrates seasonal patterns, terrain aspects, and short-term meteorological fluctuations. By contrast, vegetation indices (NDVI) showed limited predictive value in this Alpine setting, possibly because snow cover and the short growing season reduce their ecological relevance at higher elevations. Previous studies have also shown that the predictive value of NDVI may vary according to preceding meteorological conditions, particularly rainfall and humidity regimes [34], suggesting that vegetation proxies may be insufficient when considered independently from direct climatic variables.

Overall, these findings suggest that *I. ricinus* populations in this Alpine environment are maintained under conditionally suitable circumstances. While their repeated detection across years indicates persistence, the dominant role of short-term climatic drivers and the limited contribution of static environmental predictors suggest that these populations may not yet be fully stabilized. Instead, tick presence and activity at higher elevations appear to remain closely dependent on favourable and often transient environmental conditions, consistent with a transitional phase of range expansion. This interpretation aligns with recent observations from mountain systems showing that high-altitude populations may display restricted seasonal activity and stronger sensitivity to climatic variability compared with long-established lowland populations [49, 72, 73]. Moreover, our results support a system in which relatively stable site-level conditions define baseline suitability, whereas short-term meteorological variability regulates temporal fluctuations in questing activity within sites. Accordingly, although IRN presence and density did not significantly vary across years, the strong influence of short-term climatic drivers indicates that tick activity remains highly dependent on favourable environmental windows, rather than reflecting uniformly stable population dynamics.

The observed altitudinal pattern provides additional insight into these dynamics. IRN presence and density increased up to approximately 1400 m a.s.l., before declining at higher elevations. Rather than reflecting a simple negative relationship, this pattern likely results from a balance between opposing ecological constraints. Mid-altitude areas may provide optimal combinations of temperature, humidity, vegetation structure and host availability, whereas lower elevations may be affected by higher desiccation stress and higher elevations by colder temperatures, prolonged snow cover, and reduced host density [1, 74]. Similar non-linear relationships between altitude and *I. ricinus* abundance have been described in Alpine systems, where variations in local thermo-hygrometric conditions across elevation gradients explained substantial differences in tick density and activity [29, 30]. These findings suggest that the observed altitudinal pattern reflects the interaction of climatic, vegetation and host-related factors along mountain gradients.

Our findings also indicate that questing activity increased after several days of unsuitable weather conditions. Some authors stated that lagged variables outperform concurrent meteorological predictors [14, 33], whereas others observed stronger effects of concurrent conditions [18, 21, 75]. In our model, both lagged and concurrent variables contributed to IRN density, suggesting that questing activity reflects the interaction between accumulated environmental conditions and immediate microclimatic constraints. IRN density increased with relative humidity up to approximately 74%, then decreased, whereas high SD_air_ values negatively affected questing. These findings support the idea that climatic thresholds regulating questing behaviour may themselves depend on preceding weather patterns, although explicit modelling of such interactions was beyond the scope of this study.

This interpretation is further supported by the observed seasonal pattern. IRN activity showed a clear peak in spring (April-June) and no consistent autumnal peak. While this differs from the bimodal phenology commonly described in temperate European regions, it is consistent with unimodal patterns reported in northern and high-altitude areas, where questing activity is concentrated in late spring and early summer [76]. Such patterns have been associated with climatic constraints, including shortened favourable seasons and, in some contexts, drought or precipitation regimes that may suppress or blur the autumn peak [77, 78]. In Alpine environments, the short vegetation season and early onset of unfavourable conditions may limit late-season activity of nymphs, while the modest increase observed in October–November 2016 suggests that interannual variability in weather conditions may occasionally extend the questing period [79]. Overall, these observations support the interpretation of a system in which tick activity remains closely tied to transient environmental conditions rather than strictly recurring seasonal patterns.

Wildlife presence, estimated through environmental traces, was not retained in the final model. This likely reflects both measurement limitations and the indirect role of hosts in influencing questing behaviour. Differences in detectability across habitats probably contributed to measurement noise, resulting in an indicator that only partially reflects actual wildlife activity. Moreover, host-related effects may operate more strongly on long-term population maintenance and dispersal, whereas questing dynamics are more directly constrained by short-term microclimatic conditions [20]. These findings therefore suggest that host-related variables may be difficult to capture accurately using indirect field indicators and may become partially overshadowed when more proximal environmental drivers are simultaneously modelled.

We modelled the spatial process as stationary and treated space and time as separable. Nonetheless, an autoregressive temporal term was incorporated at the site level, assuming that high IRN density in one transect would be followed by comparably high density in subsequent transects at the same location. This assumption is consistent with the observed heterogeneity among sampling sites and the non-independence arising from repeated observations, as indicated by the GLMMs, and therefore justified accounting for temporal dependence within each site. The meteorological data available for the model were also constrained by the limited number of weather stations. Although temperature values were adjusted for altitude, other variables, such as wind and rainfall, were relatively uniform across the study area. To minimize the influence of extreme events and local anomalies, we used the proportion of days unsuitable for questing activity of nymphs (i.e., do-chill30d, do-dry30d, do-wind7d) rather than raw meteorological measurements. Similar concerns have been noted by Estrada-Peña et al. [37], who emphasised that studies of tick ecology often face challenges related to spatial autocorrelation and multicollinearity among environmental predictors.

To improve model interpretability and avoid over-reliance on fully parameterised models containing numerous partially correlated environmental predictors, we adopted a two-step approach. First, variable selection was guided by prior ecological knowledge and parsimony criterion, favouring synthetic variables and reducing redundancy among candidate predictors. Second, Bayesian LASSO regression was used as a complementary variable-selection framework to identify the most stable and informative predictors within a relatively high-dimensional ecological setting. Rather than being employed solely to address multicollinearity, the penalised Bayesian approach allowed us to distinguish broad environmental associations from the subset of variables that consistently contributed to explaining variation in IRN density, thereby improving the ecological interpretability of the model. Site-level clustering and non-independence arising from repeated observations, as indicated by the GLMMs, were incorporated into the model through an autoregressive term at site level. Temporal dependence was also considered but limited to the preceding transect, rather than extending across years as performed in other studies [32]. This choice reflects the dynamic environmental conditions typical of Alpine ecosystems, where abrupt weather changes, shortened warm seasons, and oscillations between excessive humidity and dryness can rapidly alter tick activity [80].

This study has several limitations. Sampling was preferential and constrained by accessibility, resulting in uneven coverage of environmental conditions and limiting the representativeness of some variables, such as slope exposure. In addition, meteorological data were derived from a limited number of weather stations, reducing spatial resolution. Wildlife presence was estimated indirectly and should therefore be interpreted cautiously. These constraints limit the generalizability of the results beyond the study area.

Despite these limitations, the study provides important insights into the ecological processes shaping *I. ricinus* populations in Alpine environments. By jointly modelling broad environmental descriptors and fine-scale climatic variables, our findings suggest that these predictors capture complementary aspects of the same ecological gradient. Within this framework, fine-scale climatic variables provided greater explanatory power for IRN density, supporting a more mechanistic interpretation of tick distribution in mountainous landscapes. More broadly, the results suggest that recently established Alpine tick populations may persist under dynamically regulated and condition-dependent ecological circumstances, highlighting the importance of integrating high-resolution climatic information into future ecological surveillance and risk assessment frameworks for tick-borne diseases.

## Conclusions

This study highlights the role of fine-scale climatic and microenvironmental conditions in shaping the distribution and activity of *I. ricinus* within a recently colonized Alpine environment. Although IRN populations were consistently detected across years, our findings indicate that high altitude persistence does not necessarily correspond to fully stabilised ecological establishment. Instead, tick presence and questing activity at higher elevations appear to remain strongly dependent on transient favourable climatic conditions, supporting the interpretation of dynamically regulated populations operating near environmental suitability limits.

By integrating multi-year field observations with meteorological and remote-sensing variables, this study extends previous Alpine investigations beyond the description of broad ecological associations. In particular, our results suggest that broader ecological descriptors of environmental suitability such as altitude, habitat type, and wildlife presence may represent complementary indicators of the same underlying environmental gradient operating at different spatial and temporal scales. Within the Bayesian framework, fine-scale climatic variables were preferentially retained, indicating that they provide a more detailed characterization of the conditions associated with questing activity of *I. ricinus* in mountainous systems. These findings are consistent with ongoing climate- and land-use-driven shifts in tick distribution across Alpine regions. However, they also indicate that the presence of *I. ricinus* at higher elevations should not automatically be interpreted as evidence of fully stabilised mountain populations. Instead, Alpine tick populations may persist under conditionally suitable circumstances, where short-term meteorological variability continues to play a major role in regulating seasonal activity and local persistence.

More broadly, this study demonstrates the importance of considering both broad environmental proxies and proximal climatic drivers when interpreting vector expansion dynamics in heterogeneous mountain ecosystems. In this context, the Bayesian LASSO framework proved useful not only for handling collinearity among ecological predictors, but also for jointly modelling the relative contribution of proximal climatic drivers and broad environmental proxies within recently colonised Alpine systems. Continued integration of long-term field surveillance, high-resolution environmental data, and flexible modelling approaches will be essential to better understand the dynamics of Alpine tick populations and to anticipate future changes in tick-borne disease risk under ongoing environmental change.

## Supplementary Information

Additional file 1.

## Data Availability

Data supporting the main conclusions of this study are included in the manuscript.
