# Supplementary material for: From valleys to peaks: how climate and environment drive Alpine populations of Ixodes ricinus
Source: Parasit Vectors. 2026 Aug 4;19:470. doi: 10.1186/s13071-026-07590-z (PMC13726165; doi:10.1186/s13071-026-07590-z)
Supplement: Supplementary file 1 [file 13071_2026_7590_MOESM1_ESM.docx]

**Tab. S1** *I. ricinus* presence and density across months.

| **Month** | **N. transects** | **Prop. of positive transects (95%CI)** | **Average *I. ricinus* / 100m^2^ (95%CI)** |
| --- | --- | --- | --- |
| Apr | 66 | 74.2% (62.6–83.3) | 5.2 (3.3–8.1) |
| May | 138 | 58.7% (50.4–66.6) | 4.5 (3.3–6.1) |
| Jun | 156 | 57.1% 49.2–64.6) | 4.2 (3.1–5.6) |
| Jul | 143 | 42.0% (34.2–50.2) | 2.5 (1.8–3.4) |
| Aug | 127 | 29.9% (22.6–38.4) | 1.2 (0.9–1.8) |
| Sep | 127 | 43.3% (35.0–52.0) | 1.1 (0.8–1.6) |
| Oct | 83 | 42.2% (32.1–52.9) | 1.5 (1.0–2.3) |
| Nov | 32 | 15.6% (6.9–31.8) | 0.2 (0.1–0.5) |

[95%CI: 95% confidence interval]

**Tab. S2** *I. ricinus* presence and density among years.

| **Month** | **N. transects** | **Prop. of positive transects (95%CI)** | **Average *I. ricinus* / 100m^2^ (95%CI)** |
| --- | --- | --- | --- |
| 2016 | 336 | 42.6% (37.4–47.9) | 2.7 (2.2–3.4) |
| 2017 | 208 | 51.4% (44.7–58.1) | 3.4 (2.6–4.5) |
| 2018 | 219 | 49.8% (43.2–56.3) | 2.5 (1.9–3.2) |
| 2019 | 109 | 48.6% (39.4–57.9) | 2.1 (1.4–3.1) |

[95%CI: 95% confidence interval]

**Tab. S3** *I. ricinus* presence and density across altitudinal ranges.

| **Altitude** | **N. transects** | **Prop. of positive transects (95%CI)** | **Average *I. ricinus* / 100m^2^ (95%CI)** |
| --- | --- | --- | --- |
| 950–1200 m | 407 | 48.8% (44.1–53.7) | 2.9 (2.4–3.4) |
| 1201–1400 m | 137 | 72.3% (64.2–79.1) | 5.2 (3.9–6.9) |
| 1401–1600 m | 142 | 66.9% (58.8–74.1) | 3.4 (2.6–4.6) |
| 1601–1800 m | 97 | 15.5% (9.6–24.0) | 0.2 (0.1–0.4) |
| > 1800 m | 89 | 4.5% (1.8–11.0) | 0.04 (0.01–0.13) |

[95%CI: 95% confidence interval]

**Tab. S4** *I. ricinus* presence and density among habitat types.

| **Habitat** | **N. transects** | **Prop. of positive transects (95%CI)** | **Average *I. ricinus* / 100m^2^ (95%CI)** |
| --- | --- | --- | --- |
| Deciduous woods | 114 | 75.4% (66.8–82.4) | 7.8 (5.6–10.8) |
| Coniferous woods | 441 | 53.3% (48.6–57.9) | 2.7 (2.3–3.2) |
| Open areas | 317 | 28.7% (24.0–33.9) | 1.0 (0.8–1.2) |

[95%CI: 95% confidence interval]

**Tab. S5** *I. ricinus* presence and density by finding of animal traces.

| **Traces** | **N. transects** | **Prop. of positive transects (95%CI)** | **Average *I. ricinus* / 100m^2^ (95%CI)** |
| --- | --- | --- | --- |
| No traces | 252 | 44.0% (40.0–48.1) | 2.3 (2.0–2.8) |
| Livestock | 12 | 50.0% (25.4–74.6) | 1.3 (0.4–4.3) |
| Wildlife | 154 | 53.7% (47.9–59.3) | 3.6 (2.8–4.6) |

[95%CI: 95% confidence interval]

**Tab. S6** Descriptive statistics of the covariates included in the Bayesian LASSO model.

| **Variable** | **Unit** | **Range** | **Average ± standard deviation** |
| --- | --- | --- | --- |
| Altitude | m a.s.l. | 959–1884 | 1327.4 ± 290.8 |
| Wildlife traces | {0, 1} | 0-1 | 0.3 ± 0.2 |
| Slope | % | 1.1–72.6 | 28.8 ± 17.5 |
| SD_air_ | mmHg | 0.2–54.0 | 9.1 ± 6.6 |
| SD7d | mmHg | 0.0–8.3 | 3.7 ± 1.7 |
| RHground | % | 20.2–100.0 | 61.5 ± 16.5 |
| Rainfall7d | mm/m^2^ | 0.0–91.6 | 11.6 ± 16.4 |
| Do–wind7d | % | 0.0–14.3 | 0.5 ± 2.6 |
| Do–chill30d | % | 0.0–56.7 | 3.3 ± 8.4 |
| Do–dry30d | % | 0.0–3.3 | 0.1 ± 0.6 |
| GHI30d | kWh/m^2^ | 0.4–8.2 | 5.5 ± 1.8 |
| NDVIavg | [–1, +1] | -0.4–0.9 | 0.6 ± 0.2 |
| NDVIlate–spring | [–1, +1] | -0.01–0.8 | 0.5 ± 0.2 |

**Fig. S1** Heatmap of pairwise Spearman correlations among the covariates included in the Bayesian LASSO model. Correlations are scale-independent, and variables were not standardised.

**Tab. S7** Generalized variance inflation factors (GVIF) and degrees-of-freedom standardized GVIF for the saturated GLMM model for IRN presence.

|  | **GVIF** | **Df** | **GVIF^(1/2×Df))^** |
| --- | --- | --- | --- |
| Altitude | 1.395511 | 1 | 1.181318 |
| Animal presence | 1.250493 | 2 | 1.057475 |
| Slope | 1.067036 | 1 | 1.032974 |
| SD_air_ | 3.222632 | 1 | 1.795169 |
| SD_7d_ | 3.437873 | 1 | 1.854150 |
| RH_ground_ | 1.637119 | 1 | 1.279499 |
| Rainfall_7d_ | 1.452579 | 1 | 1.205230 |
| do-wind_7d_ | 1.196108 | 1 | 1.093667 |
| do-chill_30d_ | 2.669436 | 1 | 1.633841 |
| do-dry_30d_ | 1.260287 | 1 | 1.122625 |
| GHI_30d_ | 11.289347 | 1 | 3.359962 |
| NDVI_avg_ | 4.109067 | 1 | 2.027083 |
| NDVI_late-spring_ | 1.459829 | 1 | 1.208234 |
| Sampling session | 21.070428 | 6 | 1.289158 |

**Tab. S8** Generalized variance inflation factors (GVIF) and degrees-of-freedom standardized GVIF for the saturated GLMM model for IRN presence.

|  | **GVIF** | **Df** | **GVIF^(1/2×Df))^** |
| --- | --- | --- | --- |
| Altitude | 1.253056 | 1 | 1.119400 |
| Animal presence | 1.276656 | 2 | 1.062964 |
| Slope | 1.030407 | 1 | 1.015090 |
| SD_air_ | 2.835160 | 1 | 1.683793 |
| SD_7d_ | 3.224175 | 1 | 1.795599 |
| RH_ground_ | 1.422391 | 1 | 1.192641 |
| Rainfall_7d_ | 2.093759 | 1 | 1.446983 |
| do-wind_7d_ | 1.072601 | 1 | 1.035664 |
| do-chill_30d_ | 4.721057 | 1 | 2.172799 |
| do-dry_30d_ | 1.338307 | 1 | 1.156852 |
| GHI_30d_ | 6.359093 | 1 | 2.521724 |
| NDVI_avg_ | 5.475707 | 1 | 2.340023 |
| NDVI_late-spring_ | 1.249158 | 1 | 1.117657 |
| Sampling session | 21.087867 | 6 | 1.289247 |

**Tab. S9** Moran’s I computed on the residuals of the saturated GLMM model for IRN presence.

| **k-nearest neighbours** | **Moran’s I** | **Standardize deviate** | **p-value** |
| --- | --- | --- | --- |
| 1 | -0.01786775 | 0.028908 | 0.489 |
| 2 | 0.09211926 | 0.88762 | 0.1874 |
| 3 | 0.02726536 | 0.48683 | 0.3132 |
| 4 | -0.002616218 | 0.23429 | 0.4074 |
| 5 | 0.010370081 | 0.43841 | 0.3305 |
| 10 | -0.017841340 | 0.11182 | 0.455 |

**Fig. S2** Variogram of the residuals of the saturated GLMM model for IRN presence.

**Fig. S3-A** Autocorrelation function (ACF) of residuals of the saturated GLMM model for IRN presence by site 1-16.

**Fig. S3-B** Autocorrelation function (ACF) of residuals of the saturated GLMM model for IRN presence by site 17-32.

**Fig. S3-C** Autocorrelation function (ACF) of residuals of the saturated GLMM model for IRN presence by site 33-44.

**Fig. S4-A** Partial autocorrelation function (PACF) of residuals of the saturated GLMM model for IRN presence by site 1-16.

**Fig. S4-B** Partial autocorrelation function (PACF) of residuals of the saturated GLMM model for IRN presence by site 17-32.

**Fig. S4-C** Partial autocorrelation function (PACF) of residuals of the saturated GLMM model for IRN presence by site 33-44.

**Tab. S10** Akaike Information Criteria (AIC) for the saturated GLMM for IRN presence both with and without autoregressive term of lag 1 (AR1).

| **Model** | **Df** | **AIC** | **Log-likelihood** |
| --- | --- | --- | --- |
| Model without AR1 | 22 | 712.2727 | -334.1363 |
| Model with AR1 | 23 | 713.3546 | -333.6773 |

[Likelihood ratio test: 0.9181164, Df = 1, p-value = 0.3379700]

**Tab. S11** Moran’s I computed on the residuals of the saturated GLMM model for IRN density.

| **k-nearest neighbours** | **Moran’s I** | **Standardize deviate** | **p-value** |
| --- | --- | --- | --- |
| 1 | 0.20151219 | 1.1918 | 0.1167 |
| 2 | 0.31114309 | 2.5425 | 0.005502 |
| 3 | 0.24146320 | 2.5212 | 0.005848 |
| 4 | 0.179063496 | 2.27 | 0.01161 |
| 5 | 0.159293875 | 2.3526 | 0.009322 |
| 10 | 0.057610602 | 1.6517 | 0.0493 |

**Fig. S5** Variogram of the residuals of the saturated GLMM model for IRN density.

**Fig. S6-A** Autocorrelation function (ACF) of residuals of the saturated GLMM model for IRN density by site 1-16.

**Fig. S6-B** Autocorrelation function (ACF) of residuals of the saturated GLMM model for IRN density by site 17-32.

**Fig. S6-C** Autocorrelation function (ACF) of residuals of the saturated GLMM model for IRN density by site 33-44.

**Fig. S7-A** Partial autocorrelation function (PACF) of residuals of the saturated GLMM model for IRN density by site 1-16.

 **Fig. S7-B** Partial autocorrelation function (PACF) of residuals of the saturated GLMM model for IRN density by site 17-32.

**Fig. S7-C** Partial autocorrelation function (PACF) of residuals of the saturated GLMM model for IRN density by site 33-44.

**Tab. S12** Akaike Information Criteria (AIC) for the saturated GLMM for IRN density both with and without autoregressive term of lag 1 (AR1).

| **Model** | **Df** | **AIC** | **Log-likelihood** |
| --- | --- | --- | --- |
| Model without AR1 | 22 | 2996.584 | -1476.292 |
| Model with AR1 | 23 | 2744.314 | -1349.157 |

[Likelihood ratio test: 2.542702×10^2^, Df = 1, p-value = 3.044643×10^-57^]

**Tab. S13** Estimated coefficients, Monte-Carlo standard errors (MC s.e.), and inclusion probabilities (γ) from the Bayesian LASSO model across a range of shrinkage levels (δ).

|  | **δ = 0.05** | | | **δ = 0.1** | | | **δ = 0.2** | | | **δ = 5.0** | | | **δ = 20.0** | | |
| --- | --- | --- | --- | --- | --- | --- | --- | --- | --- | --- | --- | --- | --- | --- | --- |
| **Variable** | **Coeff.** | **MC s.e.** | **γ** | **Coeff.** | **MC s.e.** | **γ** | **Coeff.** | **MC s.e.** | **γ** | **Coeff.** | **MC s.e.** | **γ** | **Coeff.** | **MC s.e.** | **γ** |
| Wildlife | 0.03 | 0.02 | 0.36 | -0.08 | 0.46 | 0.48 | 0.02 | 0.69 | 0.21 | 0.02 | 0.04 | 0.21 | 0.03 | 0.40 | 0.36 |
| Do-chill_30d_ | 0.59 | 0.01 | 1 | 0.60 | 0.11 | 1 | 0.67 | 0.16 | 1.00 | 0.67 | 0.02 | 1.00 | 0.59 | 0.12 | 1.00 |
| Do-dry_30d_ | -0.01 | 0.03 | 0.16 | -0.02 | 0.71 | 0.12 | -0.00 | 0.71 | 0.18 | -0.00 | 0.04 | 0.18 | -0.01 | 0.56 | 0.16 |
| GHI_30d_ | 0.66 | 0.02 | 1.00 | 0.80 | 0.18 | 1.00 | 0.77 | 0.20 | 1.00 | 0.77 | 0.03 | 1.00 | 0.66 | 0.12 | 1.00 |
| Rainfall_7d_ | 0.01 | 0.02 | 0.20 | -0.03 | 0.58 | 0.17 | 0.04 | 0.62 | 0.18 | 0.04 | 0.03 | 0.18 | 0.01 | 0.46 | 0.20 |
| Altitude | 0.02 | 0.02 | 0.32 | -0.03 | 0.57 | 0.36 | 0.02 | 0.72 | 0.18 | 0.02 | 0.03 | 0.18 | 0.02 | 0.49 | 0.32 |
| Slope | -0.02 | 0.03 | 0.35 | 0.37 | 0.12 | 1.00 | 0.07 | 0.67 | 0.20 | 0.07 | 0.03 | 0.20 | -0.02 | 0.47 | 0.35 |
| NDVI_late spring_ | -0.36 | 0.05 | 0.64 | 0.42 | 0.32 | 0.88 | 0.12 | 0.63 | 0.45 | 0.12 | 0.03 | 0.45 | -0.36 | 0.46 | 0.64 |
| RH_ground_ | -0.21 | 0.00 | 1.00 | -0.21 | 0.05 | 1.00 | -0.21 | 0.05 | 1.00 | -0.21 | 0.00 | 1.00 | -0.21 | 0.05 | 1.00 |
| NDVI_avg_ | 0.02 | 0.02 | 0.26 | 0.01 | 0.66 | 0.21 | 0.13 | 0.63 | 0.40 | 0.13 | 0.04 | 0.40 | 0.02 | 0.47 | 0.26 |
| SD_air_ | -0.45 | 0.01 | 1.00 | -0.47 | 0.07 | 1.00 | -0.46 | 0.06 | 1.00 | -0.46 | 0.01 | 1.00 | -0.45 | 0.07 | 1.00 |
| Do-wind_7d_ | 0.10 | 0.00 | 1.00 | 0.10 | 0.02 | 1.00 | 0.10 | 0.02 | 0.99 | 0.10 | 0.00 | 0.99 | 0.10 | 0.02 | 1.00 |
| Habitat: coniferous wood | 0.02 | 0.02 | 0.42 | -0.03 | 0.73 | 0.39 | 0.07 | 0.62 | 0.38 | 0.07 | 0.03 | 0.38 | 0.02 | 0.42 | 0.42 |
| Habitat: deciduous wood | -0.03 | 0.02 | 0.39 | 1.43 | 0.43 | 1.00 | 0.89 | 0.70 | 0.89 | 0.89 | 0.09 | 0.89 | -0.03 | 0.47 | 0.39 |
| Habitat: open areas | 0.05 | 0.04 | 0.46 | -0.04 | 0.57 | 0.43 | -0.22 | 0.78 | 0.52 | -0.22 | 0.07 | 0.52 | 0.05 | 0.48 | 0.46 |
| SD_7d_ | 0.02 | 0.02 | 0.24 | 0.03 | 0.70 | 0.22 | -0.04 | 0.64 | 0.20 | -0.04 | 0.03 | 0.20 | 0.02 | 0.49 | 0.24 |
